# Perineal endometriosis on an episiotomy scar: diagnosis based on clinical, radiological, and hormonal criteria (case report)

**DOI:** 10.11604/pamj.2025.51.25.48002

**Published:** 2025-05-28

**Authors:** Abdoulrazak Egueh Nour, Chirwa Mahamoud Abdillahi, Samia Bennani, Ahmed Hared Bouh, Bouknani Nawal, Amal Rami

**Affiliations:** 1 Department of Radiology, Cheikh Khalifa International University Hospital, Mohammed VI University of Sciences and Health (UM6SS), Casablanca, Morocco,; 2 Department of Pediatrics, Cheikh Khalifa International University Hospital, Mohammed VI University of Sciences and Health (UM6SS), Casablanca, Morocco

**Keywords:** Cyclical perineal pain, episiotomy scar, perineal endometriosis, hormonal therapy, case report

## Abstract

Endometriosis is a chronic, non-cancerous gynecological disorder that is typically observed in women of fertile age. It describes the presence of functional endometrial components, glands and stroma outside the endometrial cavity, most often involving pelvic structures such as the ovaries, peritoneum, and uterine ligaments. Perineal endometriosis is a rare form of extrapelvic endometriosis, with an estimated incidence of between 0.3% and 1%. We report the case of a 36-year-old woman, gravida 1 para 1(G1P1), with a history of vaginal delivery and no known history of endometriosis, who presented with a painful mass located on the right perineal scar two years after undergoing a mediolateral episiotomy. The mass was associated with cyclical pain and significantly impaired quality of life. Clinical examination, combined with ultrasound and Magnetic Resonance Imaging (MRI), suggested the diagnosis of perineal endometriosis in the absence of other endometriotic lesions. As the patient declined surgery, hormonal therapy was initiated, resulting in marked symptom improvement at 8-month follow-up. Although histological confirmation was not obtained, the typical clinical presentation, evocative MRI features, and favorable response to hormonal therapy supported the diagnosis of perineal endometriosis. This under-recognized entity should be systematically considered in women of reproductive age presenting with cyclical perineal pain following episiotomy.

## Introduction

Endometriosis is a relatively frequent condition in women of reproductive age, with a reported prevalence of 10% to 20% [1]. It is characterized by the presence of functional endometrial tissue outside the uterine cavity, most commonly within the pelvic region. Extrapelvic locations, however, are rare, particularly perineal endometriosis affecting only 0.3% and 1% of women [2]. The classic diagnostic clinical trial for this condition includes a history of vaginal delivery with episiotomy, increasing cyclical perineal pain and a perineal mass located at the episiotomy scar [3]. The pathogenesis of perineal endometriosis remains unclear despite numerous theories. The leading hypothesis proposes that endometrial cells are directly implanted onto scar tissue following obstetric trauma [4].

## Patient and observation

**Patient information:** a 36-year-old woman, gravida 1 para 1, with no known history of endometriosis, presented with a progressive right perineal mass localized at the site of a mediolateral episiotomy performed two years earlier during vaginal delivery.

**Clinical findings:** the patient reported cyclical perineal pain worsening during menstruation, without associated signs of inflammation, infection, or urinary symptoms. Several consultations with gynecologists over the previous months had failed to identify a cause, and symptoms significantly impacted her quality of life. Clinical examination revealed a firm, tender, non-inflammatory nodule at the episiotomy scar, with healthy surrounding skin and no fistula or discharge.

**Timeline of current episode:** the patient began experiencing right perineal pain six months before consultation, progressively worsening and becoming cyclical. Imaging investigations were performed eight months after symptom onset, leading to the initiation of hormonal therapy with a favorable clinical response.

**Figure 1:**
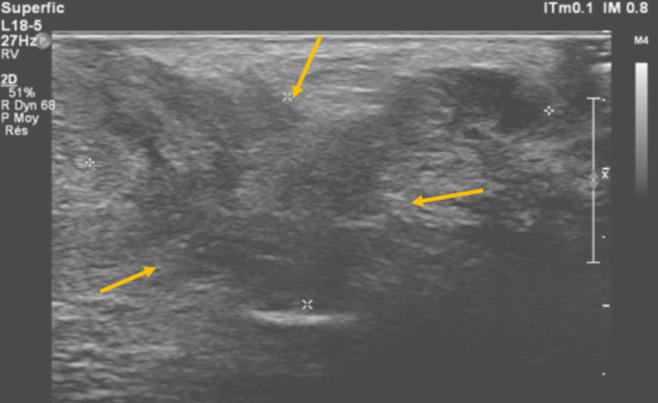
transperineal ultrasound revealed a heterogeneous hypoechoic mass in the right perineum, with retractile and irregular contours measuring 32x15mm (yellow arrows)

**Figure 2:**
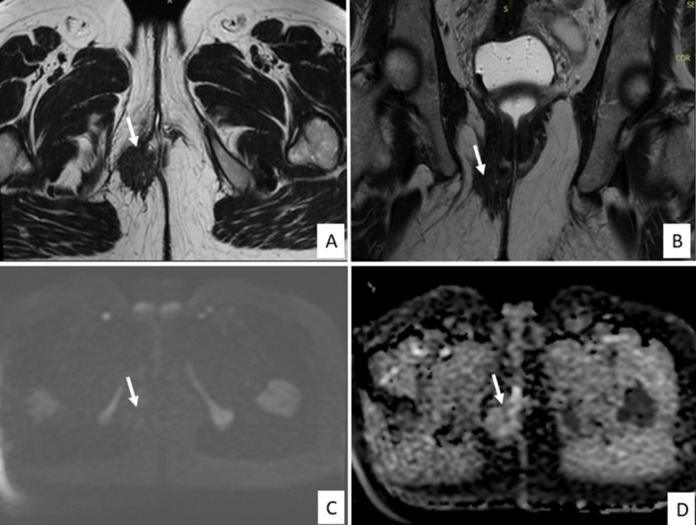
axial (A) and coronal (B) T2-weighted MR images demonstrating a right-sided perineal mass with irregular, stellate contours extending toward the posterolateral wall of the distal third of the vagina and infiltrating the subcutaneous fat; The lesion exhibits low signal intensity on T2-weighted sequences, interspersed with small hyperintense cystic foci; no diffusion restriction is observed on DWI and ADC images (C, D)

**Figure 3:**
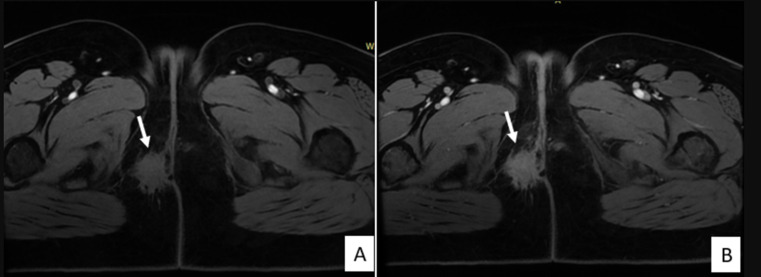
on the T1-weighted fat-suppressed sequence (A); the lesion appears isointense relative to skeletal muscle, containing small hyperintense foci; Axial post-contrast T1 fat-saturated image showing no enhancement after gadolinium injection (B)

**Figure 4:**
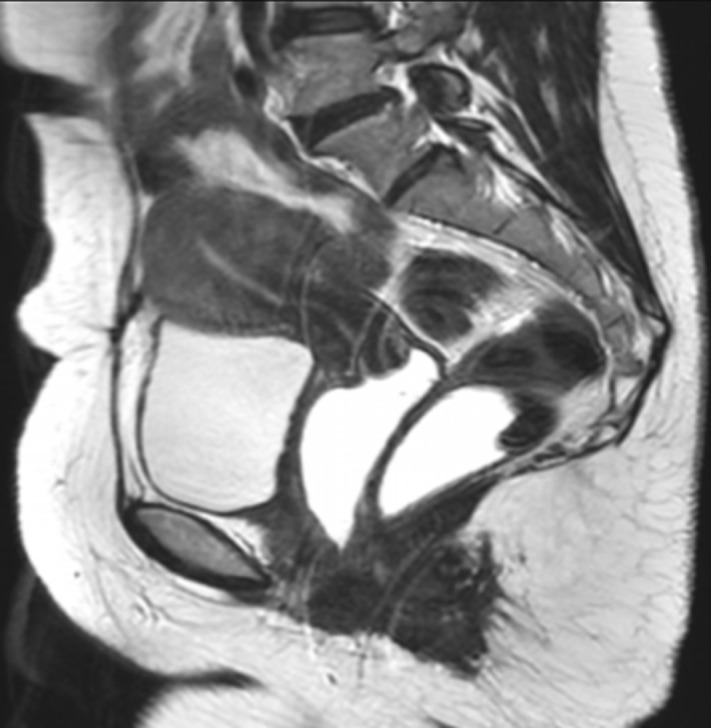
the sagittal T2-weighted sequence revealed no evidence of deep infiltrating endometriosis or adenomyosis

**Diagnostic assessment:** high-resolution ultrasound revealed a 32 x 15 mm hypoechoic, heterogeneous, retractile mass within the episiotomy scar, with no color Doppler flow (Figure 1). Pelvic MRI showed a perineal mass with spiculated, retractile margins, isointense to muscle on T2-weighted images, containing focal T1 hyperintense areas on fat-suppressed sequences, and no contrast enhancement or diffusion restriction. The lesion measured 4 cm in height and 4 x 2 cm in axial diameter (Figure 2 and Figure 3). The uterus, ovaries, rectum, and bladder appeared normal, without signs of deep pelvic endometriosis (Figure 4). Laboratory tests, including inflammatory markers, were normal.

**Diagnosis:** based on the typical clinical triad (cyclical pain, history of episiotomy, localized mass), characteristic imaging findings, and exclusion of alternative diagnoses, perineal endometriosis was strongly suspected.

**Therapeutic interventions:** the patient declined surgical management and opted for hormonal therapy with progestins.

**Follow-up and outcome of interventions:** clinical response was favorable, with significant improvement in symptoms and partial regression of the mass on physical examination. At the 8-month follow-up, no recurrence or complications were observed.

**Patient perspective:** the patient expressed satisfaction with the non-invasive management and relief of symptoms, and was informed of the possibility of recurrence and the need for long-term monitoring.

**Informed consent:** written informed consent was obtained from the patient for publication of this case report and accompanying images.

## Discussion

Perineal endometriosis is an uncommon but well-defined form of extrapelvic endometriosis, initially described by Schickele in 1923 [3]. Its low incidence, estimated at approximately 0.06% in a retrospective study of 72 patients by Nominato *et al*. [5], and its frequent association with obstetric scars, particularly episiotomies, make it a singular entity that deserves attention in cases of painful perineal masses in women of reproductive age. Two pathophysiological mechanisms are traditionally described to explain the occurrence of this form of endometriosis: the direct transplantation of endometrial cells during surgical intervention, such as episiotomy, referred to as secondary perineal endometriosis [6], as in our patient´s case. The second mechanism is the lymphovascular dissemination of endometrial cells in cases without prior perineal trauma, known as primary perineal endometriosis [6]. Most reported cases involve patients with a history of episiotomy or obstetric-related perineal tear, with symptom onset varying from several months to years after the procedure, with a median of 30 months. It is essential to consider perineal endometriosis as a possible diagnosis in a young, fertile woman presenting with localized pain or a mass in the ano-perineal region. A retrospective study by Zhu *et al*. conducted on 36 cases, identified three essential diagnostic criteria with a 100% predictive value when all are present [7]: a history of episiotomy or prior perineal tear, a firm and tender mass at the perineal lesion, and cyclical pain with mass swelling during menstruation (as was the case for our patient).

This clinical profile is crucial for differentiating perineal endometriosis from other differential diagnoses, such as localized recurrent ano-perineal abscess, which presents as a fluctuating and recurrent perineal mass; suture granuloma, urethral cysts, or Bartholin gland cysts, and rarer conditions such as ano-perineal melanoma, which may present similarly [8]. Malignant transformation into clear cell carcinoma should be ruled out in cases of recurrent scar endometriosis [3].

The assessment of perineal endometriosis primarily relies on imaging techniques such as ultrasound and MRI [8], with computed tomography (CT) being generally less useful. Ultrasound, though less specific, is often used as an initial modality for detecting scar endometriosis and may reveal hypoechoic or heterogeneous lesions with internal echoes with size and shape variations [7]. Magnetic resonance imaging, the reference imaging modality, is essential for diagnosing and evaluating the local extent of endometriosis, allowing precise visualization of the endometriotic mass and excluding other deep pelvic or extrapelvic locations. In MRI, endometriosis on an episiotomy scar presents several characteristic aspects that vary depending on disease stage and infiltration extent. In our case, a fibrous thickening with T2 hypointensity and stellate retractile infiltration was observed [8]. Typical MRI characteristics include T1 and T2 hyperintensity without fat saturation, suggesting the presence of endometrial tissue.

In the absence of histological confirmation, the diagnosis in our patient was supported by the convergence of the typical clinical triad, evocative MRI features, and favorable hormonal therapy response. This approach is endorsed by several authors who acknowledge that in typical presentations, diagnosis may be presumed without surgical confirmation, especially when surgery is declined or contraindicated [9].

While surgical excision with clear margins remains the standard of care to minimize recurrence risk, selected cases may benefit from medical management [3]. Progestins or GnRH analogs can offer satisfactory symptom control [10]. In our case, hormonal therapy led to a marked clinical improvement, with pain resolution and mass reduction within a few months. The sustained favorable course over 8 months further supports the presumed diagnosis.

The lack of histological confirmation and surgery remains the main limitation of this case. The diagnosis is based on strong clinical and radiological presumption and therapeutic response, without definitive exclusion of alternative etiologies. This case underscores the importance of an integrated diagnostic approach when excision is not performed.

## Conclusion

Perineal endometriosis should be suspected in women presenting with perineal pain that recurs in a cyclical pattern, particularly with a history of episiotomy or perineal tear. Early management and recognition of this rare pathology are essential to prevent potentially serious complications, such as deep invasion of adjacent structures or, in rare cases, malignant transformation. Although perineal endometriosis is rare, its management relies on strong clinical knowledge, judicious imaging use, and appropriate surgical treatment.
